# Less Polar Compounds and Targeted Antioxidant Potential (In Vitro and In Vivo) of *Codium adhaerens* C. Agardh 1822

**DOI:** 10.3390/ph14090944

**Published:** 2021-09-21

**Authors:** Sanja Radman, Ana-Marija Cikoš, Ivana Flanjak, Sanja Babić, Lara Čižmek, Drago Šubarić, Rozelindra Čož-Rakovac, Stela Jokić, Igor Jerković

**Affiliations:** 1 Department of Organic Chemistry, Faculty of Chemistry and Technology, University of Split, Ruđera Boškovića 35, 21000 Split, Croatia; sradman@ktf-split.hr; 2 Department of Process Engineering, Faculty of Food Technology, Josip Juraj Strossmayer University of Osijek, Franje Kuhača 18, 31000 Osijek, Croatia; acikos@ptfos.hr; 3 Department of Food and Nutrition Research, Faculty of Food Technology, Josip Juraj Strossmayer University of Osijek, Franje Kuhača 18, 31000 Osijek, Croatia; ivana.flanjak@ptfos.hr; 4 Laboratory for Aquaculture Biotechnology, Division of Materials Chemistry, Ruđer Bošković Institute, Bijenička cesta 54, 10000 Zagreb, Croatia; sanja.babic@irb.hr (S.B.); lara.cizmek@irb.hr (L.Č.); rozelindra.coz-rakovac@irb.hr (R.Č.-R.); 5 Department of Food Technology, Faculty of Food Technology, Josip Juraj Strossmayer University of Osijek, Franje Kuhača 18, 31000 Osijek, Croatia; dsubaric@ptfos.hr

**Keywords:** dimethyl sulfide, heptadecane, pheophytin *a* and its derivatives, pheophorbide *a* and its derivatives, radical scavenging and antioxidant power, zebrafish model

## Abstract

*Codium adhaerens* from the Adriatic Sea (Croatia) was comprehensively investigated regarding less polar compounds for the first time. Although there are several phytochemical studies on *C. adhaerens* from other regions, this is the first report on volatile organic compounds (VOCs) from fresh (FrCa) and air-dried (DrCa) samples. The novelty is also related to its targeted antioxidant potential in vitro and in vivo. The main aims were to: (a) identify and compare VOCs of FrCa and DrCa obtained by headspace solid-phase microextraction (HS-SPME) and hydrodistillation (HD); (b) determine fatty acid (FA) composition of freeze-dried sample (FdCa); (c) determine the composition of less polar fractions of FdCa by high-performance liquid chromatography–high-resolution mass spectrometry with electrospray ionisation (UHPLC-ESI-HRMS); and (d) comprehensively evaluate the antioxidant activity of the fractions by four in vitro assays and in vivo zebrafish model (including embryotoxicity). Significant changes of VOCs were found after air drying. ω6 FAs were present in higher content than ω3 FAs indicating *C. adhaerens* as a good source of dietary polyunsaturated FAs. The results obtained in vivo correlate well with in vitro methods and both fractions exerted similar antioxidative responses which is in agreement with the high abundance of present biomolecules with known antioxidant properties (e.g., fucoxanthin, pheophytin *a*, and pheophorbide *a*). These results suggest that *C. adhaerens* might be a potent source of natural antioxidants that could be further used in the research of oxidative stress-related diseases.

## 1. Introduction

Interest in using green seaweeds as natural resources has recently increased because of their bioactive constituents, which may be used for medical purposes. Green seaweeds, in general, contain lipids, proteins, peptides, polysaccharides, carotenoids, phenolic compounds, and alkaloids [1,2]. Seaweeds are known to possess nutritional benefits as food and have found much use in industry and medicine for various purposes. Their compounds have complex structures that have shown different biological activities [3,4,5], including anticancer activity, in several in vitro and in vivo models such as polysaccharides (antibacterial, anticancer, anticoagulant, anti-inflammatory, antioxidant, antiviral, hepatoprotective, immunostimulatory, others), phlorotanins (antibacterial, anti-Alzheimer’s, anticancer, antidiabetic, anti-inflammatory, antioxidant, antiviral, cytoprotective, hepatoproptective, immunomodulatory, neuroprotective, others), terpenoids (anticancer, antifungal, antioxidant, others), alkaloids (antibacterial, anticancer, anti-inflammatory, antioxidant, antiviral, neuroprotective, others), or carotenoids (anticancer, antidiabetic, anti-inflammatory, anti-obesity, antioxidant, neuroprotective, others).

A survey of the literature revealed different phytochemical studies on *Codium adhaerens*. Bioactive compounds present in its ethanol extract were analysed by gas chromatography–mass spectrometry (GC-MS) after the silylation [6]. Proline, mannitol and β-sitosterol were found. Concerning fatty acids, it was found that *C. adhaerens* contained palmitic, α-linolenic, oleic, stearic, arachidonic, eicosapentaenoic and arachidic acids. Relative pigment composition of *C. adhaerens* was determined by the cellulose thin-layer chromatogram and chlorophyll *a*, chlorophyll *b* and siphonaxanthin were determined [7]. Amino acid composition was determined before and after the hydrolysis in *C. adhaerens* ethanol extract and alanine, asparagine, aspartic acid, glutamic acid, glutamine, glycine, isoleucine, proline, serine, taurine, threonine and valine were found [8]. The sterol profiles were evaluated by the procedure involving alkaline hydrolysis and extraction followed by separation by reversed-phase high-performance liquid chromatography (HPLC)–diode array detection (HPLC-DAD) and *C. adhaerens* contained desmosterol, ergosterol, fucosterol, cholesterol, campesterol, stigmasterol and β-sitosterol [9]. Recently, a great deal of interest has been directed toward the isolation of novel sulfated polysaccharides (SPs) from marine green algae because of their numerous health-beneficial effects. Green seaweeds are known to synthesise large quantities of SPs, and arabinose was reported to be the major monosaccharide of SPs from *C. adhaerens* [2,10]. These SPs exhibit many beneficial biological activities, such as anticoagulant, antiviral, antioxidative, antitumor, immunomodulating, antihyperlipidemic and antihepatotoxic activities [2,10].

However, no reports on the volatile organic compounds (VOCs) from *C. adhaerens* were found, and therefore we decided to investigate them in detail. Since our previous studies [11,12] indicated great diversity of VOCs from fresh and air-dried samples (including *Codium bursa*) we decided to research the variability of VOCs of *C. adhaerens* from fresh (FrCa) and air-dried (DrCa) samples. In addition, VOCs have often been related to less polar constituents, whose comprehensive antioxidant potential from *C. adhaerens* (in vivo and in vitro) was targeted in the current research for the first time. The main goals of the present research were to: (a) identify and compare VOCs of FrCa and DrCa obtained by headspace solid-phase microextraction (HS-SPME) and hydrodistillation (HD) followed by analysis with gas chromatography and mass spectrometry (GC-MS); (b) determine fatty acid composition of freeze-dried sample (FdCa) after derivatisation as methyl esters by gas chromatography analysis with flame-ionisation detector (GC-FID); (c) determine the composition of less polar fractions of FdCa by high-performance liquid chromatography–high-resolution mass spectrometry with electrospray ionisation (UHPLC-ESI-HRMS); (d) comprehensively evaluate the antioxidant activity of the fractions by four in vitro assays (Folin–Ciocalteu, reduction of radical cation ABTS^•+^, 2,2-diphenyl-1-picryl-hydrazyl-hydrate (DPPH) assay, and ferric reducing antioxidant power (FRAP)) and in vivo zebrafish model (including embryotoxicity).

## 2. Results and Discussion

### 2.1. Headspace Composition

Two fibres of different polarity (divinylbenzene/carboxene/polydimethylsiloxane (DVB/CAR/PDMS) and polydimethylsiloxane/divinylbenzene (PDMS/DVB)) were used for HS-SPME. By this approach, more complete headspace profile could be achieved due to combination of the fibres of different polarities. In FrCa headspace (HS-FrCa) 91.52% of VOCs were identified in total by HS-SPME with DVB/CAR/PDMS fibre and 83.25% by HS-SPME with PDMS/DVB fibre, Table 1. 

**Figure 1:**
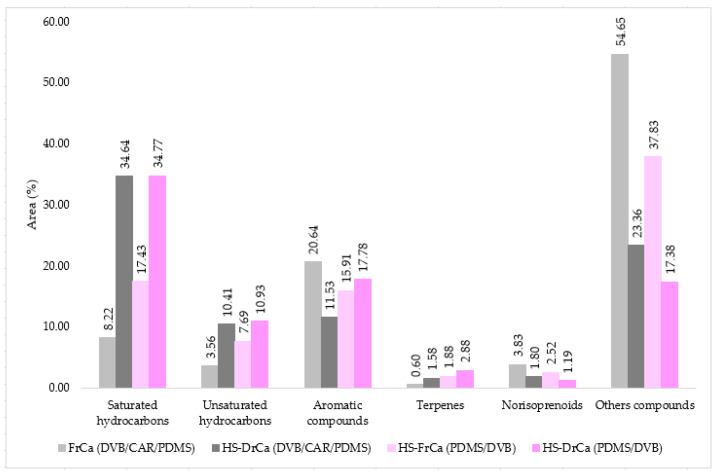
The VOCs from *C. adhaerens* sorted by structural groups extracted by HS-SPME.

In DrCa headspace (HS-DrCa), 82.08% (DVB/CAR/PDMS fibre) and 84.91% (PDMS/DVB fibre) of VOCs were identified in total by HS-SPME. The majority of identified VOCs in HS-FrCa belong to sulphur compounds, organoiodines, esters, and lactones, and in HS-DrCa the majority of VOCs were comprised of saturated hydrocarbons. The dominant compound in HS-FrCa was dimethyl disulfide, DMS, (54.25%—DVB/CAR/PDMS fibre; 37.83%—PDMS/DVB fibre). Distribution of the compound structural groups in HS-FrCa and HS-DrCa is presented in Figure 1.

The headspace chemical profile showed that DMS was the main identified compound in the seagrass *Posedonia oceanica* (59.3%), green alga *Flavellia petiolata* (22.2%), brown alga *Halopteris filicina* (12.8%) [13] and green alga *C. bursa* (56.51%) [12]. It is known that DMS can be easily oxidised into dimethyl sulfoxide (DMSO) in the contact with air [14,15] which can be noticed as the result of air-drying of *C. adhaerens*. Namely, the amount of DMS decreased 129.2 times (DVB/CAR/PDMS fibre) or 61.0 times (PDMS/DVB fibre) in the sample after air-drying, resulting in the detection of highly abundant DMSO (19.50%; 12.38%). The second dominant compound in HS-FrCa was the aromatic compound benzaldehyde (12.84%; 12.22%), which decreased 3.5 or 5.9 times in HS-DrCa (3.64%; 2.06%). The loss of benzaldehyde during air-drying could be a consequence of its higher volatility in comparison to benzyl alcohol rather than its oxidation to benzoic acid that cannot be found by HS-SPME, and was also not identified by HD. The percentage of aromatic compound benzyl alcohol, as the second most abundant compound is HS-DrCa, increased 3.0 (or 7.7) times in HS-DrCa from 2.22% (or 1.60%) to 6.71% (or 12.35%). Its increase during drying could be connected to lignin degradation, since lignin-related compounds were found in the plant cell walls of *Codium fragile* [16]. In fact, the green algae extracellular coverings, including cell walls, showed remarkable structural and biochemical similarity to the land plant cell walls containing assemblages of polymers with notable similarity to pectins, cellulose, hemicelluloses, extensin, arabinogalactan proteins (AGPs), and lignin [17]. Aliphatic hydrocarbon heptadecane was the major compound in HS-DrCa. Heptadecane is known to be the most abundant alkane in green algae [18,19]. The abundance of heptadecane in HS-DrCa was higher with DVB/CAR/PDMS fibre (26.61%) with respect to PDMS/DVB fibre (23.12%). The increment of heptadecane with air-drying was noticed (4.0 times—DVB/CAR/PDMS fibre; 2.1 times—PDMS/DVB fibre), probably as a result of fatty acid decarboxylation [20]. Additionally, two saturated aliphatic hydrocarbons were noticed to increase by a great percentage after air-drying (probably due to fatty acids degradation): tridecane (3.2 times; or 2.5 times) and pentadecane (9.1 times; or 4.9 times). In HS-DrCa, three organoiodines were identified (more abundant with PDMS/DVB fibre): iodomethane (2.00%; or 2.48%), diiodomethane (0.26%; or 0.40%) and 1-iodopentane (0.31%; or 0.90%); while in HS-FrCa none of them were detected. Marine macroalgae showed great potency of linking halogen ions resulting in halogenated secondary metabolites formation [21]. Palmer et al. [22] showed that different oxidative stress, including desiccation, increased the volatile iodinated compounds in brown alga *Laminaria digitata*. Bravo-Linares et al. [23] investigated the production of VOCs in response to environmental stresses and they have found that all tested algae showed greater release of iodinated compounds under stress, especially iodomethane. Even though the brown algae showed the greatest release of iodinated compounds (iodoethane, 2-iodopropane, 1-iodobutane, diiodomethane and iodomethane), the green algae *Ulva lactuca* and *Enteromorpha* sp. also contained iodinated compounds that increased during drying process. In the chemical profile of brown alga *Aschophyllum nodosum* headspace 1-iodopentane (0.8%) was identified as the only organoiodine [13]. It has already been indicated that the volatile halogenated compounds may be involved in the defence mechanism of many different organisms including algae [24]. The enzymes involved in halogenated organic compounds synthesis are haloperoxidases [24].

### 2.2. Volatile Oil Composition

In the hydrodistillate of FrCa (HD-FrCa) 87.16% and of DrCa (HD-DrCa) 97.96% of total ion chromatogram area were identified in total, Table 2.

**Figure 2:**
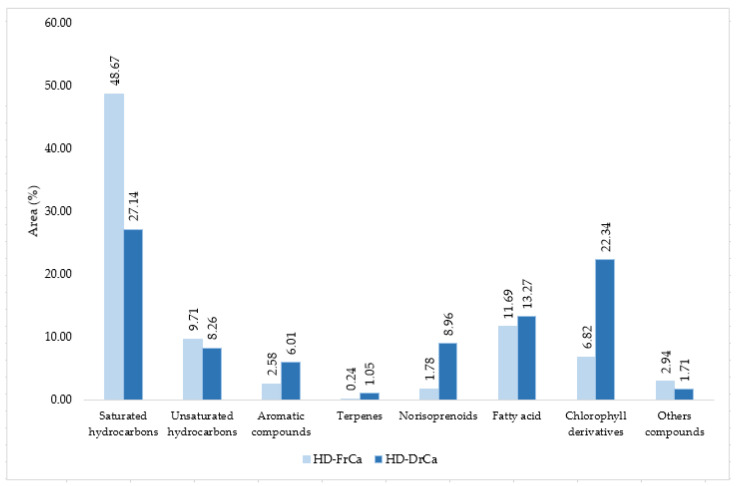
The VOCs from *C. adhaerens* obtained by HD sorted by structural groups.

The majority of the identified VOCs in both HD-FrCa and HD-DrCa belong to the group of aliphatic compounds—more precisely, saturated hydrocarbons (50.53% HD-FrCa; 27.88% HD-DrCa), Table 2. The prevalent compound in HD-FrCa was saturated hydrocarbon heptadecane (29.32%), which was found with 2.4 times lower abundance in HD-DrCa (12.29%). The origin of heptadecane was connected with direct decarboxylation of stearic acid [19]. The second most abundant saturated hydrocarbon, pentadecane, decreased 1.8 times during air-drying (HD-FrCa 3.25%; HD-DrCa 1.84%), and the most abundant unsaturated hydrocarbon, pentadec-1-ene, decreased 3.2 times after air-drying (HD-FrCa 2.12%; HD-DrCa 0.67%). Although hexadecane was previously found in *C. bursa* among the major constituents [12], pentadecane was found with a small percentage only in air-dried *C. bursa* hydrodistillate. The group of chlorophyll derivatives, as the second most represented group (22.80%), and carotenoid degradation products (norisoprenoids) (7.03%) showed significant increase in HD-DrCa. The major photosynthetic pigments of green algae are chlorophyll *a* and *b* [25]. Phytol, acyclic diterpene alcohol, is linked to the chlorophyll forming ester linkage [26]. The percentage of (*E*)-phytol increased from 5.00% in HD-FrCa to 16.99% in HD-DrCa, indicating chlorophyll degradation. The percentage of phytone, the oxidation product of phytol, increased from 2.03% in HD-FrCa to 5.81% in HD-DrCa. Jerković et al. [12] reported a great increase of phytol and phytone in hydrodistillate of air-dried *C*. *bursa*. Five norisoprenoids were identified in HD-FrCa: C_11_-norisoprenoid (β-cyclohomocitral), and C_13_-norisoprenoids (3,4-dehydroionene, hexahydropseudoionone, α-ionone and β-ionene). The oxidative cleavage of carotenoids leads to the formation of norisoprenoid compounds. Due to differences in the length of carotenoid precursor chain and possible positions of the cleavage, C_9_- to C_13_-norisoprenoids could be formed [27]. Hence, in HD-DrCa, two C_10_-norisoprenoids (safranal and β-cyclocitral) and one C_13_-norisoprenoid (β-ionone) were identified. α-Ionone and β-ionene showed the percentage increase during air-drying (probably due to the oxidation): α-ionone (0.61% in HD-FrCa; 5.84% in HD-DrCa) and β-ionene (0.85% in HD-FrCa; 1.81% in HD-DrCa). Even though hydrodistillation is not very adequate for long chain fatty acids determination (since they are semivolatile or nonvolatile) several fatty acids were detected: dodecanoic (C12:0) acid (0.52% in HD-FrCa; 0.19% in HD-DrCa), tetradecanoic (C14:0) acid (2.43% in HD-FrCa; 0.24% in HD-DrCa), hexadecanoic (C16:0) acid (8.62% in HD-FrCa; 10.55% in HD-DrCa) and (9*Z*)-octadec-9-enoic (C18:1n9*c*) acid (0.49% in HD-FrCa; 2.53% in HD-DrCa). Distribution of the compound structural groups in HD-FrCa and HD-DrCa is presented in Figure 2.

### 2.3. Fatty Acid Composition

A total of 13 fatty acids were identified in freeze dried *C. adhaerens* (FdCa), Table 3. The most abundant fatty acids (FAs) were palmitic (C16:0) and arachidic (C20:0) acids with average values 25.50% and 22.48%, respectively. 

Pereira et al. [28] and Andrade et al. [6] also confirmed the dominance of the mentioned fatty acids in marine green algae from *Codium* species. Generally, the total saturated fatty acid (SFA) content (64.94%) was higher than the content of unsaturated fatty acids (UFAs), which was 35.51% (24.66% for monounsaturated FAs (MUFAs) and 10.85% for polyunsaturated FAs (PUFAs)). The prevalence of SFAs over UFAs in *Codium* species has been reported previously [6,28,29]. Oleic acid isomers (C18:1n9*c*+ C18:1n9*t*) were the main unsaturated fatty acids (16.91%) followed by palmitoleic acid (C16:1) and *cis*-linoleic acid (C18:2n6*c*) whose content was 5.79% and 4.67%, respectively. Although SFAs are dominant in analysed *C. adhaerens*, ω6 FAs were presented in higher content than ω3 FAs (Table 3). Higher ω6 FAs than ω3 FAs content was found in a previous study [9] for *C. bursa*. PUFAs are well known for their positive impact on human health. The importance is even higher if the ω6 FAs: ω3 FAs ratio in food is between 1.5 and 3 that is generally accepted as the balance value for human nutrition [30]. Considering that the ω6 FAs: ω3 FAs ratio obtained in this study was 2.92, marine green alga *C. adhaerens* can be considered a good source of dietary PUFAs.

### 2.4. Less Polar Non-Volatile Compounds from F3 and F4 Fractions

**Figure 3:**
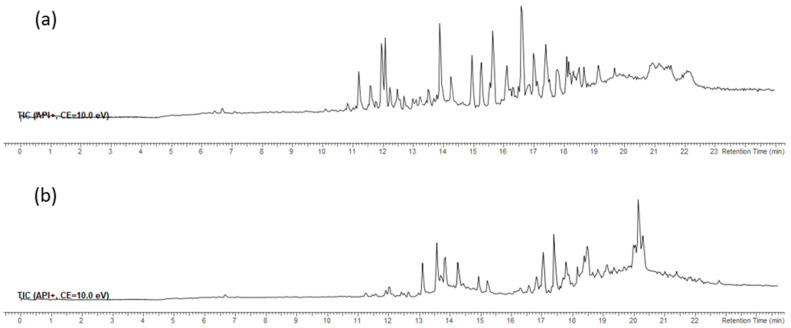
Total ion chromatogram (TIC) of the fractions (**a**) F3 and (**b**) F4.

**Figure 4:**
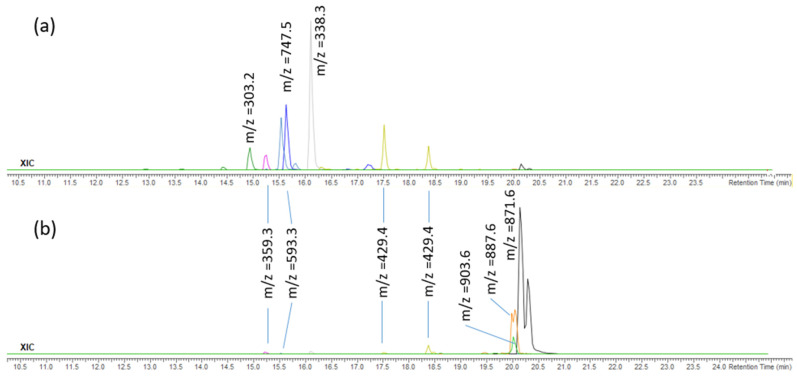
Extracted ion chromatograms (XIC) of most abundant ions in the fractions (**a**) F3 and (**b**) F4 (zoomed).

The FdCa sample was fractionated (Section 3.7) to obtain less polar fractions F3 and F4, which were analysed by high-performance liquid chromatography–high-resolution mass spectrometry with electrospray ionisation (UHPLC-ESI(+)-HRMS). The major compounds (in terms of signal intensity) from the obtained chromatograms in positive ion mode were tentatively identified on the basis of their elemental compositions and tandem mass spectra (Table 4), and they belong to: chlorophyll derivatives, fatty acid glycerides and related compounds, terpenes, steroids, and carotenoids. Total ion chromatograms of the fractions F3 and F4 are shown in Figure 3 and extracted ion chromatograms (XIC) of most abundant ions in the fractions F3 and F4 are shown in Figure 4.

Well-known green pigment chlorophyll was not detected in the fractions, but its derivatives devoid of magnesium atoms constituted the major compounds, particularly in F4 (Table 4). Identified chlorophyll derivatives can be divided into two sub-groups containing 55 or 35 (without the aliphatic side chain) carbon atoms. The first subgroup includes four highly lipophilic compounds with significant abundance mainly in F4, except for the compound **18** (Table 4), which was also present in F3, since it is more polar due to the additional keto group. The main component of this sub-group is pheophytin *a*, which has previously been found in notable amounts in different macroalgae [31]. Pheophytin *a* has been identified from edible green alga *Enteromorpha prolifera*, and showed a potent anti-inflammatory activity [32], and has been reported to be a potent suppressive substance against *umu* C gene expression in a tester bacteria induced by genotoxic substances [33]. In addition, previous studies have reported that pheophytin *a* and pheophytin *a*-related compounds show antioxidant activity in the autooxidation of lipids [34,35]. The other three compounds of this subgroup were pheophytin *a* derivatives characterised by the presence of an additional double bond, carbonyl, or hydroxyl group in their composition. The second subgroup consisted of three compounds (Table 4) having the common structure (pheophorbide *a* and its derivatives differ from pheophytin *a* by the absence of the long side hydrocarbon chain). Pheophorbide *a* was much more abundant in F3 than in F4. It is a photosensitiser that can induce significant anti-proliferative effects in several human cancer cell lines [36]. 

Fatty acid glycerides in F3 and F4 consisted of monoglycerides of palmitic, stearic, oleic, and arachidonic acids that were found prevailing in *C. adhaerens* fatty acid composition (Table 3). Three diglycerides (one of stearic acid and two of oleic acid) were found (Table 4). Three other compounds, chemically related to this group, were identified as fatty amide (13-docosenamide), fatty acid esters (palmitate and stearate) of 2-hydroxypropanol. Sugar fatty acid ester (octadecatrienoate) and diester of octadecatrienoic and hexadecatrienoic acids were also found. One glycosylmonoacylglycerol (gingerglycolipid A) was present. In addition, the high molecular derivative of 4-oxobutanoic acid with elemental composition C_44_H_80_N_2_O_17_ was identified (Table 4, compound **14**).

Terpenes and steroids comprised five compounds (Table 4). The tandem mass spectrum of C_20_H_32_O_2_ showed two H_2_O molecules loss and specific carbon backbone fragmentation pattern that permitted assigning diterpenoid isoamijiol structure, similar to *F. virsoides* [8]. Previously, it was isolated from brown alga *Dictyota linearis* [37]. Another compound (more abundant in F3) with a similar retention time and the same molecular formula C_20_H_30_O_2_ containing only one hydroxyl was tentatively identified as isoamijiol oxidation product possessing keto-group. Two major steroids (Table 4) were identified as monool (β-stigmasterol) and (3β)-3-hydroxystigmast-5-en-7-one possessing hydroxy and keto groups.

Among xanthophyll carotenoids, only fucoxanthin was found in F3 and F4. It has been reported as the main carotenoid pigment in all brown algae [11,38] that possess different biological activities, i.e., antioxidant and anticancer [39,40].

### 2.5. Antioxidant Activity of F3 and F4 Fractions In Vitro

**Figure 5:**
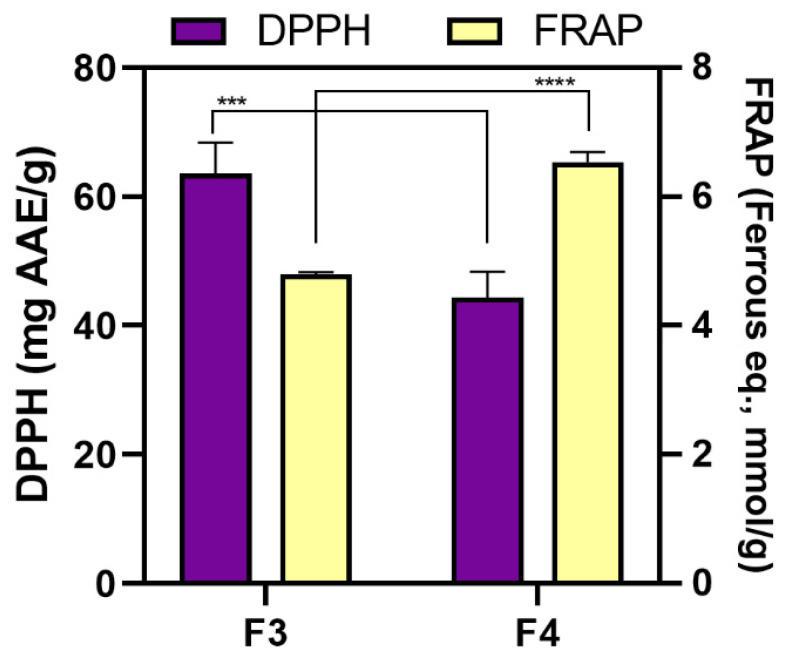
Antioxidant activity of *C. adhaerens* less polar fractions (F3 and F4) obtained using 2,2-diphenyl-1-picryl-hydrazyl-hydrate (DPPH) and ferric reducing antioxidant power (FRAP) in vitro assays (mean ± SD; *n* = 3). An asterisk indicates a significant difference between F3 and F4 (*** *p* < 0.01; **** *p* < 0.001).

Within this study, in vitro evaluation of the antioxidant activity of two less polar fractions F3 and F4 obtained from *C. adhaerens* was performed using four different spectroscopic methods: Folin–Ciocalteu (F–C), FRAP, DPPH, and ABTS assays. Although F–C assay is commonly known as a measure of total phenolic content, here it represents a rate of an overall antioxidant activity since the extracts do not contain phenols (as in our previous study on *F. virsoides* [11]). The results obtained using F–C assay indicated 3-fold higher (*p* < 0.0001) activity of F4 (64.27 ± 0.73 mg GAE/g F4) then F3 (21.12 ± 0.91 mg GAE /g F3). Interestingly, although the obtained values correspond to the values from our two previously published studies on *Amphiroa rigida* [41] and *Fucus virsoides* [11], one peculiarity can be observed. In *C. adhaerens*, higher activity was observed for F4, which is not the case in the other two macroalgae where higher activity is obtained in F3. This aberration could be explained by the different chemical compositions of *C. adhaerens* fractions (Table 4). The antioxidant activity of F3 and F4 was further tested by implementing additional two antioxidant assays—DPPH and FRAP, as depicted in Figure 5. Although DPPH implies dominant reaction through single electron transfer (SET), DPPH radical can also react through transfer of hydrogen atom (HAT), while FRAP assay is only based on SET. The inhibition percentage using DPPH assay for a tested concentration of 1 mg/mL for both fractions was around 20%. Nevertheless, when normalised per gram of the fraction, F3 (59.69 ± 2.32 mg AAE/g fraction) showed slightly higher (*p* < 0.005) antioxidant activity then F4 (44.34 ± 4.05 mg AAE/g fraction). However, the reverse result was obtained when conducting FRAP analysis. Higher activity (*p* < 0.0001) was obtained for F4 (6.54 ± 0.15 mmol ferrous eq./g fraction), then F3 (4.80 ± 0.04 mmol ferrous eq./g fraction). In F4, the dominant compound is pheophytin *a* along with its derivatives which are known for their antioxidant ability [42], while in F3 pheophytin *a* is not detected. This is in accordance with the results obtained using FRAP assay, where higher activity was observed in F4, thus indicating easier reaction through single electron transfer probably due to pheophytin *a* and its side chain in the molecular structure. Conversely, the results obtained using DPPH assay showed relatively low antioxidant activity of both fractions, but slightly higher activity for F3 could be explained by the presence of pheophorbide *a* (PPB*a*), which is also a known product related to the chlorophyll with already proven biological activity [36]. Sudha et al. [43] evaluated the antioxidant activity of *C. adhaerens* by implementing soxhlet extraction with ethyl acetate as solvent. The obtained extract showed the highest activity against free DPPH radical at the concentration of 1000 μg/mL with an inhibition percentage of around 70%.

Additionally, the reduction of radical cation by implementing ABTS assay was also evaluated. To obtain the IC_50_ curve, different concentrations of F3 and F4 were prepared ranging from 0.05 to 5 mg/mL, followed by increasing the inhibition percentage. IC_50_ values for both F3 and F4 were calculated as shown in Table 5 with the corresponding confidence interval, slope and coefficient of determination (R^2^). As can be seen, the IC_50_ value for both fractions is almost identical and is around 2.4 mg/mL, which represents the higher concentration responsible for the inhibition of 50% of free radicals. This could be explained by the dominant presence of different pigments, i.e., the presence of PPB*a* in F3 and pheophytin *a* in F4 with the addition of carotenoid fucoxanthin that exhibits antioxidant activity [44] and is present in both fractions in a similar ratio. Pinteus et al. [45] also analysed the antioxidant activity of numerous algae, including *C. adhaerens*, but with an emphasis on more polar compounds. They also observed relatively low antioxidant activity with IC_50_ values for both methanolic and dichloromethane extracts higher than 1000 μg/mL [45]. The inhibition percentage using ABTS assay for tested concentration of 1 mg/mL for both fractions was around 40% (data not shown).

### 2.6. Developmental Toxicity of F3 and F4 Fractions in Zebrafish Embryo

To evaluate the potential toxicity of tested *C. adhaerens* fractions on embryonic development of zebrafish, mortality, morphological changes and hatchability at 96 hpf were analysed. Upon exposure to 500, 250 and 125 µg/mL of F3 and F4, no statistically significant changes in survival were observed (*p* < 0.05; data not shown). Additionally, no significant morphological abnormalities or hatching inhibition were seen in the tested concentration range. Mortality in the control groups (negative control (AW) and solvent controls (1% MeOH, 1% DMSO)) was <5%. Given the obtained results, concentrations of 500, 250 and 125 µg/mL were selected for further experiments.

### 2.7. Protective Effects of F3 and F4 Fractions against H_2_O_2_-Induced Oxidative Stress

Although valuable, in vitro methods do not reflect physiological processes like absorption and metabolism of antioxidants, and therefore a compound that shows good antioxidant properties within in vitro tests may not necessarily be biologically active. For that reason, zebrafish *Danio rerio* embryos were employed for this research, as currently one of the most perspective vertebrate model organisms [46]. To determine whether *C. adhaerens* fractions play a role in ROS-mediated oxidative stress, zebrafish embryos were exposed to F3 and F4 fractions in the presence of H_2_O_2_. A statistically significant increase in the mortality rate was observed in the H_2_O_2_-treated group (66.7 ± 5.8%). However, pre-treatment with the highest tested concentration (500 µg/mL) of both, F3 and F4, declined mortality rates by 23.3% and 26.7%, respectively, compared to H_2_O_2_-treated group. Lower concentrations (125 µg/mL and 250 µg/mL) of both fractions had no impact on zebrafish survival.

**Figure 6:**
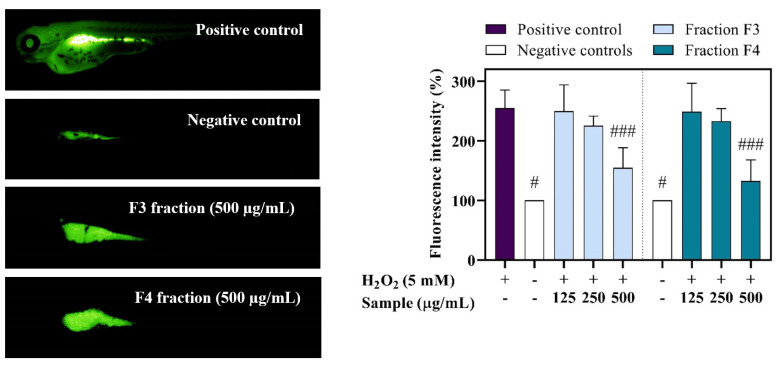
ROS scavenging potential of *C. adhaerens* fractions F3 and F4 in zebrafish. Left: Representative fluorescence images of H_2_O_2_ treated and the fractions co-treated zebrafish larvae. Right: Mean fluorescent intensity of DCF in the whole larvae calculated using Image J program. Sign # represents significant difference when compared with H_2_O_2_-treated group (# *p* < 0.05; ### *p* < 0.001). Each treatment was normalised relative to non-treated controls (100%).

ROS production was significantly elevated by H_2_O_2_ treatment (255.9%) when compared to control groups (normalised to 100%). A concentration-dependent reduction in the DCF fluorescence intensity was observed in the specimens preincubated with the highest concentration of the fractions (Figure 6). The treatment with 500 µg/mL of F3 and F4 resulted in significant decrease of ROS generation to 154.8% (*p* < 0.001) and 132.7% (*p* < 0.001), respectively.

Such findings imply that tested fractions protect cells against H_2_O_2_-induced oxidative stress, ultimately leading to a decrease in zebrafish mortality. When compared, the results obtained using the zebrafish in vivo model correlate well with the ones obtained with in vitro methods. According to both used methods, F3 and F4 exerted similar antioxidative responses which is in agreement with the high abundance of the biomolecules in the fractions with already proven antioxidant properties. Specifically, fucoxanthin, pheophytin *a*, and pheophorbide *a* have attracted extensive interest due to their beneficial biological activities including anti-cancer, anti-inflammatory, anti-oxidant, anti-angiogenic, and anti-wrinkle activity [47,48,49]. Kang et al. [44] demonstrated the protective effects of fucoxanthin (25–100 µM) against high glucose-induced oxidative damage in a zebrafish model. Even though antioxidant activities of phaeophytin *a* and pheophorbide *a* were not determined using zebrafish model, in vitro methods (DPPH, TBARS assay) proved their strong antioxidant potential [48]. Moreover, the reduction of free radicals and the protection of cultured lymphocytes against oxidative DNA damage was observed in the presence of those two chlorophyll *a* derivatives [48,50]. For that reason, we assume that the high abundance of pheophytin *a* in F4 contributed to the higher antioxidant potential.

When compared with the results published in our previous study on *Fucus virsoides* F3 and F4 fractions [8], the antioxidant potential of *C. adhaerens* was notably lower. For that reason, one should note that the antioxidant activity of each tested fraction cannot be related to the particular compound due to possible synergistic/antagonistic interactions between bioactive compounds. Thus, we strongly encourage further studies to revealthe mechanisms behind the functionality of antioxidant mixtures that can ultimately result in implementation in the food, cosmeceutical, and pharmaceutical industry.

## 3. Materials and Methods

### 3.1. Chemicals

The fatty acids methyl esters (FAMEs) were purchased from Supelco Co. (Bellefonte, PA, USA). The standards of L-ascorbic acid (≥99%), gallic acid (>97.5%), DPPH (2,2-diphenyl-1-picrylhydrazyl), TPTZ (2,4,6-tripyridyl-S-triazine, ≥98%), ABTS (diammonium salt of 2,2′-azino-bis(3-ethylbenzthiazolin-6-yl)sulfonic acid, >99.0%), and dichloro-dihydro-fluorescein diacetate (≥97%, DCF-DA) were purchased from Sigma-Aldrich (St. Louis, MO, USA).

Organic solvents (dimethyl sulfoxide (DMSO, p.a.), ethanol (p.a.), methanol (p.a.)), hydrochloric acid (HCl, p.a.), iron (III) chloride (FeCl_3_, p.a.), Folin–Ciocalteu reagent and NaHCO_3_ (p.a.) were obtained from Kemika (Zagreb, Croatia), while potassium persulfate (>98%) was purchased from Scharlab (Barcelona, Spain). Hydrogen peroxide (H_2_O_2_, 30%) was obtained from Alkaloid Skopje (Skopje, North Macedonia).

Acetonitrile with 0.1% (*v*/*v*) formic acid and water with 0.1% (*v*/*v*) formic acid, both hypergrade for HPLC-MS LiChrosolv^®^, were purchased from Supelco Co. (Bellefonte, PA, USA).

Zebrafish *D. rerio* adults of wild-type WIK strain were obtained from the European Zebrafish Resource Center of the Karlsruhe Institute of Technology (KIT), Karlsruhe, Germany.

Used solvents were of HPLC grade and were obtained from J.T. Baker (Bridgewater, NJ, USA).

### 3.2. Macroalga Sample

*Codium adhaerens* C. Agardh 1822 was collected in November 2020 by a single-point collection from the Adriatic Sea (Poluotok Rtina/Paška vrata) with the sampling geographical coordinates 44°19′14″ N; 15°55′42″ E. The sea depth was 1–3 m with the sea temperature at 15 °C. An air-tight plastic bag containing seawater and collected alga was transported to the laboratory immediately after the collection and was kept in the dark at 4 °C for not more than 48 h until further analysis. A part of the collected sample of *C. adhaerens* was placed in the dark at the room temperature for 14 days for air-drying. Both fresh and air-dried samples were cut in small pieces before further analysis.

A part of *C. adhaerens* was freeze-dried for the procedures in the Section 3.5 and Section 3.6. Before the freeze-drying, the sample was washed in water (5 times) and in deionised water (2 times), then it was cut in 5–10 mm slices and frozen at −60 °C in an ultra-low freezer (CoolSafe PRO, Labogene, Lillerød, Denmark) for 24 h. The freeze-drying was performed under a high vacuum (0.13–0.55 hPa) at −30 °C and 20 °C as the primary and secondary drying temperatures for 24 h.

### 3.3. Headspace Solid-Phase Microextraction (HS-SPME)

A part of the fresh sample was placed between two filter paper layers for few minutes to remove a part of the excess seawater. HS-SPME was performed with PAL Auto Sampler System (PAL RSI 85, CTC Analytics AG, Schlieren, Switzerland) using two SPME fibres covered with DVB/CAR/PDMS (divinylbenzene/carboxen/polydimethylsiloxane) or PDMS/DVB (polydimethylsiloxane/divinylbenzene). Both fibres were purchased from Supelco Co. (Bellefonte, PA, USA) and were conditioned prior to the extraction. Prepared samples (1 g) were placed into 20 mL glass vials sealed with stainless steel cap with polytetrafluorethylene (PTFE)/silicon septa. The method was set to equilibrate the sample at 60 °C for 15 min and then extract the sample for 45 min. The injector temperature was set to 250 °C and the thermal desorption directly to the GC column was carried out for 6 min. HS-SPME was performed in triplicate.

### 3.4. Hydrodistillation (HD)

Hydrodistillation (HD) was performed in a modified Clevenger apparatus for 2 h. Pentane (Fluka, Merck KGaA, Darmstadt, Germany) and diethyl ether (J.T. Baker Inc., Bridgewater, NJ, USA) were used as the solvent trap in *v*/*v* ratio 1:2 (1 mL). The prepared samples of fresh and air-dried *C. adhaerens* were used separately for HD. The volatile oil dissolved in the solvent trap was removed with a pipette, passed through the layer of MgSO_4_ in a small glass funnel and slowly concentrated by the slow flow of nitrogen until the volume of 0.2 mL. 2 µL were used for GC-MS analyses.

### 3.5. Gas Chromatography Mass Spectrometry Analysis of VOCs

The GC-MS analyses of isolated VOCs were carried out with an Agilent Technologies (Palo Alto, Santa Clara, CA, USA) gas chromatograph model 8890 equipped with a mass spectrometer detector model 5977E MSD (Agilent Technologies). The VOCs separation was achieved on HP-5MS capillary column (30 m × 0.25 mm, 0.25 µm film thickness, Agilent Technologies, Palo Alto, Santa Clara, CA, USA). The GC conditions and the detailed procedure are described in our previously published papers [11,12,13]: the injector and detector temperatures were 250 °C and 300 °C and the oven temperature was set up isothermal at 70 °C for 2 min. Temperature gradient was achieved increasing the temperature from 70–200 °C at 3 °C/min then was held isothermally at 200 °C for 15 min. Split ratio was 1:50; carrier gas was helium (He at flow rate 1.0 mL/min). The MSD (EI mode) was operated at 70 eV, and the mass range was set from 30 to 300 amu. The identification of the compounds was based on the comparison of their retention indices (RI), determined relative to the retention times of *n*-alkanes (C_9_–C_25_), with those reported in the literature (National Institute of Standards and Technology) and their mass spectra with the spectra from Wiley 9 (Wiley, New York, NY, USA) and NIST 17 (D-Gaithersburg) mass spectral libraries. The percentage composition of the samples was calculated using the normalisation method (without correction factors). The average component percentages in Table 1 and Table 2. were calculated from GC–MS analyses of three replicates.

### 3.6. Gas Chromatography Flame-Ionisation Detection Analysis of Fatty Acids

Total lipids were extracted from freeze dried *C. adhaerens* sample (FdCa) using Folch method [51] with chloroform/methanol (2:1 *v*/*v*). The detailed methodology of lipid extraction and preparation of FAMEs was described in our previous paper [11]. The separation of prepared FAMEs was performed on a Shimadzu GC-2010 Plus gas chromatograph equipped with a flame ionisation detector (FID) and fitted with an SH-FAMEWAX^TM^ capillary column (30 m, 0.32 mm ID and 0.25 µm df). The injector and detector temperatures were set at 240 °C and 250 °C, respectively. The injection volume was 2 μL with a split ratio of 1:100. The GC oven program was as follows: 120 °C hold for 5 min, to 220 °C at 5 °C/min, hold for 20 min. Nitrogen was used as carrier gas, flowing at the constant flow rate of 1.26 mL/min. The identification of separated FAMEs was achieved based on the comparison of retention times with the retention times of certified reference standard (Supelco F.A.M.E. Mix, C4–C24, St. Louis, MO, USA) analysed under the same conditions. The results were expressed as the percentage of identified fatty acid on total fatty acids (%).

### 3.7. Fractionation by Solid-Phase Extraction (SPE)

The freeze-dried *C. adhaerens* (FdCa) was extracted (10 mL/g solvent:solid ratio) three times with sonication (ultrasound-bath Elma, Elmasonic P 70 H, Singen, Germany; 37 kHz/50 W) for 5 min applying methanol:dichloromethane (MeOH/DCM, 1:1, *v*/*v*). The obtained extract was evaporated under nitrogen (5.0, Messer, Zapresic, Croatia), and was mixed with C18 powder (40–63 µm, Macherey-Nagel Polygoprep 60-50 C18, Fisher Scientific, Hampton, NH, USA). The obtained dry extract was then placed on an SPE cartridge (C18, particle size 40 µm, bed weight 1g, column capacity 6 mL, Agilent Bond Elut, Waldbronn, Germany), which was previously conditioned with MeOH and ultrapure water. Then the sample was eluted by applying the solvents of decreasing polarity to obtain the fractions F1 to F4 as was done in our previous paper [11]: F1 (with H_2_O), F2 (with H_2_O/MeOH (1:1, *v*/*v*)), F3 (with MeOH), and F4 (with MeOH/DCM (1:1, *v*/*v*)). Targeted less polar compounds were eluted in F3 and F4 and were dried by SpeedVac (SPD1030, Thermo Scientific, Waltham, MA, USA) and stored at 4 °C in dark.

### 3.8. Ultra High-Performance Liquid Chromatography–High-Resolution Mass Spectrometry (UHPLC-ESI-HRMS) of F3 and F4

The UPLC-HRMS analyses were performed using an ExionLC AD system (AB Sciex, Concord, Canada) equipped with the ExionLC solvent delivery system, ExionLC AD Pump, ExionLC AD Degasser, ExionLC AD Column oven, ExionLC AD Autosampler and ExionLC Controller combined with quadrupole-time-of-flight (Q-TOF) mass spectrometer TripleTOF 6600+ (AB Sciex, Concord, Canada) with Duospray ion source. The analytical column used for chromatographic separations was Acquity UPLC BEH Phenyl-Hexyl, 2.1 mm × 100 mm, particle size 1.7 µm (Waters, Milford, MA, USA). The column oven temperature was set at 30 °C and the flow rate was set at 0.4 mL/min. The mobile phases were water (A) and acetonitrile (B) both containing 0.1% formic acid. After 0.6 min of isocratic condition with 2% of B, the elution program was applied as follows: 0.6–18.5 min (B linear gradient to 100%), 18.5–25 min (100% B). The injection volume was 4 µL.

Mass spectrometry detection was conducted in the positive electrospray ionisation (ESI^+^). Tandem (MS/MS) mass spectra were recorded using collision-induced dissociation (CID) in information-dependent acquisition (IDA) mode for precursor ions with the signal intensities above 200 cps threshold. The maximum number of precursor ions simultaneously subjected to CID was 15. The ion source parameters were: nebulising gas (air, gas 1) pressure 40 psi, heater gas (air, gas 2) pressure 15 psi, curtain gas (nitrogen) pressure 30 psi, ESI capillary voltage 5.5 kV and the source temperature 300 °C. The recording mass spectra parameters were: declustering potential 80 V, *m*/*z* range 100–1000 (MS) and 20–1000 (MS/MS), and accumulation time 100 ms. The collision gas was nitrogen with the collision energy 40 eV with a spread of 20 eV. The mass scale calibrations (in the MS and MS/MS modes) were done prior to each run in an automatic regime using a Tuning Solution (AB Sciex, Concord, Canada).

The data were processed using ACD/Spectrus Processor 2021.1.0. (ACD/Labs, Toronto, Canada). The elemental compositions of the compounds were determined based on the accurate masses of the corresponding protonated molecules, their isotopic distributions, and the product ions *m*/*z* in MS/MS spectra. The tentative identification of detected components was carried out on the basis of their elemental compositions, tandem mass spectra and search in the ChemSpider database with a further selection of hits matching with MS/MS data.

### 3.9. Antioxidant Activity of Tested Fractions by In Vitro Assays

In vitro determination of antioxidant activity in this study employed four methods, including Folin–Ciocalteu method, ferric reducing antioxidant power (FRAP), reduction of the radical cation (ABTS) and 2,2-diphenyl-1-picryl-hydrazyl-hydrate (DPPH) assay. The measurements were carried out using a UV/Vis microplate reader (Infinite M200 PRO, TECAN, Männedorf, Switzerland) in multi-well plates (96-well) in triplicate. The results for all assays are expressed as mean ± standard deviation (*n* = 3). All four mentioned methods were conducted in accordance with our previous research [11]. Briefly, obtained fractions were tested for their antioxidant activity by the reactions with appropriate reagents, incubated for a known period and change in colour with regards to control or blank sample was measured. Additionally, IC_50_ curve was obtained for both F3 and F4 by implementing the ABTS assay.

### 3.10. Zebrafish Embryotoxicity Test (ZET)

A breeding stock of healthy mature wild-type strain zebrafish *Danio rerio* (original supplier: European Zebrafish Resource Center of the Karlsruhe Institute of Technology (KIT), Karlsruhe, Germany) was used within this research. Zebrafish maintenance and embryo production was described in the paper of Babić et al. (2021) [52].

ZET was performed in accordance with OECD Test Guideline, in detail described in our previous study [11]. The fractions were tested in three concentrations (500, 250 and 125 µg/mL) using 10 embryos in three replicates. Final solvent concentration (F3: MeOH; F4: DMSO) did not exceed 1%. As a negative control artificial water was used, while 1% of MeOH and DMSO was tested as the solvent control. At 96 h of exposure, mortality and abnormality rates were recorded using an inverted microscope (Olympus CKX41) equipped with Leica EC3 digital camera and LAS EZ 3.2.0 digitising software.

### 3.11. Antioxidant Effect of Tested Fractions Using Zebrafish Model

The evaluation of the protective effect of F3 and F4 against H_2_O_2_-induced oxidative stress was conducted following the protocol described in our previous study [11]. Briefly, since no toxicity was recorded when performing the ZET test, zebrafish embryos were pre-treated with F3 and F4 in the concentration of 500, 250 and 125 µg/mL for 2 h. After pre-treatment, oxidative stress was initiated with the addition of 5 mM H_2_O_2_. Upon 96 h of the exposure, the mortality rate was recorded, and survived specimens were stained with 10 μM of fluorogenic dye dichloro-dihydro-fluorescein diacetate (DCF-DA) [11]. Intracellular ROS level in zebrafish larvae was visualised using a fluorescent microscope (Olympus^®^ BX51 light binocular microscope; Microsoft^®^ AnalySIS Soft Imaging System Software) with a green fluorescent filter. The fluorescence intensity of images was quantified using ImageJ software.

Statistical analysis and graphical representation were performed using GraphPad Prism version 8.0.1 for Windows, GraphPad Software, San Diego, CA, USA, www.graphpad.com accessed on 26 August 2021. One-way analysis of variance (ANOVA) and Tukey’s post hoc test were performed to examine the significance between negative control and tested samples, as well as among treatments. When the assumption for normality was violated the Kruskal–Wallis one-way analysis of variance on ranks was performed. Student’s paired t-test was chosen to analyse the data obtained with in vitro assays. The results were expressed as means ± SD, and *p* ≤ 0.05 was used as a cut-off value of statistical significance throughout the paper.

## 4. Conclusions

*C. adhaerens* from the Adriatic Sea (Croatia) was comprehensively investigated regarding less polar compounds for the first time. Although there are several phytochemical studies of *C. adhaerens* from other regions, this is the first report on the volatile organic compounds (VOCs) from both fresh (FrCa) and air-dried (DrCa) samples. Great variability among HS-FrCa and HD-FrCa, as well as among HS-DrCa and HD-DrCa, was noted, as was expected according to the previous studies on different algae. This variability is the consequence of different applied methods isolating more and less volatile compounds as well as air-drying. However, the composition of HD and HS was partially similar to our previous study on *C. bursa*, indicating the same major VOCs (DMS and heptadecane) as chemical markers of *Codium* sp. DMS was the major compound in HS-FrCa. The oxidation of DMS to DMSO was noticed during air-drying. In HS-DrCa the majority of the identified compounds belong to alkanes with heptadecane as dominant. In HS-DrCa, three organoiodines were identified: iodomethane, diiodomethane and 1-iodopentane confirming the release of iodinated compounds under oxidative stress. The majority of VOCs in HD-FrCa and HD-DrCa belong to aliphatic compounds. The prevalent compound in HD-FrCa was heptadecane, which decreased in HD-DrCa. Both chlorophyll derivatives and carotenoid degradation products (norisoprenoids) showed significant increase in HD-DrCa. The percentage of (*E*)-phytol and its oxidative product phytone increased during air-drying, indicating chlorophyll degradation. In HD-DrCa, two C_10_-norisoprenoids (safranal and β-cyclocitral) and one more C_13_-norisoprenoid (β-ionone) were identified, confirming oxidative cleavage of carotenoids and further cleavage of norisoprenoid compounds.

Considering the ω6 FAs: ω3 FAs ratio, *C. adhaerens* can be considered a good source of dietary PUFAs. The ω6 fatty acids were present in higher content than ω3 fatty acids, as was found in previous studies, and the most abundant fatty acids were palmitic and arachidic acids.

The less polar fractions F3 and F4 were analysed by (UHPLC-ESI(+)-HRMS) for the first time. The major identified compounds were less polar compounds: chlorophyll derivatives (two sub-groups contacting 55 or 35 carbon atoms, mainly pheophytin *a*, pheophorbide *a* and their derivatives), fatty acid glycerides, terpenes, steroids, and carotenoids.

The novelty of the present research is also related to in vitro and in vivo research on targeted antioxidant potential of less polar compounds from *C. adhaerens*. When compared, the results obtained by in vivo correlate well with in vitro methods and both fractions exerted similar antioxidative responses which is in agreement with the presence of a high abundance of biomolecules with known antioxidant properties (e.g., fucoxanthin, pheophytin *a*, and pheophorbide *a*). The results obtained using in vitro and in vivo approaches showed that the tested F3 and F4 fractions, in particular the chemical composition of *C. adhaerens* from the Adriatic Sea, exhibited high antioxidant activity and protective effects against H_2_O_2_-induced mortality of zebrafish embryos. These results suggest that *C. adhaerens* might be a potent source of natural antioxidants that could be used in research of oxidative stress-related diseases associated with the excess generation of reactive oxygen species such as cancer, cardiovascular and neurodegenerative diseases and therefore further research of this alga is encouraged.

## Figures and Tables

**Table 1:** The volatile compounds from *Codium adhaerens* isolated by headspace solid-phase microextraction (HS-SPME) and analysed by gas chromatography–mass spectrometry (GC-MS): (I—fresh *C. adhaerens* extracted by DVB/CAR/PDMS fibre, II—air-dried *C. adhaerens* extracted by DVB/CAR/PDMS fibre, III—fresh *C. adhaerens* extracted by PDMS/DVB fibre, IV—air-dried *C. adhaerens* extracted by PDMS/DVB fibre).

No.	Compound	RI	Area (%) ± SD			
			I	II	III	IV
1	Dimethyl sulfide	<900	54.25 ± 2.13	0.42 ± 0.03	37.83 ± 1.19	0.62 ± 0.03
2	Iodomethane	<900	−	2.0 ± 0.07	−	2.48 ± 0.09
3	2-Ethylfuran	<900	4.52 ± 0.18	−	−	−
4	Pentanal	<900	−	0.20 ± 0.07	−	0.11 ± 0.04
5	Hexanal	<900	0.65 ± 0.03	1.67 ± 0.09	2.46 ± 0.93	2.59 ± 0.36
6	Dimethylsulfoxide	<900	−	19.50 ± 0.49	−	12.38 ± 0.62
7	Heptanal	902	−	0.95 ± 0.16	0.90 ± 0.35	2.29 ± 0.37
8	Diodomethane	923	−	0.26± 0.01	−	0.40 ± 0.10
9	1-Iodopentane	927	−	0.31 ± 0.04	−	0.902 ± 0.21
10	α-Pinene	941	−	0.25 ± 0.05	−	0.73 ± 0.21
11	(2*E*)-Hept-2-enal	964	−	−	−	0.86 ± 0.30
12	Benzaldehyde	966	12.84 ± 0.50	3.64 ± 0.04	12.22 ± 5.62	2.06 ± 0.13
13	Oct-1-en-3-ol	985	2.45 ± 0.10	1.95 ± 0.01	2.40 ± 0.68	1.27 ± 0.11
14	Phenol	987	−	−	0.64 ± 0.08	1.20 ± 0.17
15	6-Methylhept-5-en-2-one	989	0.63 ± 0.02	0.64 ± 0.01	0.43 ± 0.16	0.63 ± 0.01
16	2-Pentylfuran	996	1.07 ± 0.04	0.90 ± 0.00	0.58 ± 0.22	1.80 ± 0.20
17	Octanal	1004	−	0.75 ± 0.05	0.73 ± 0.28	1.15 ± 0.13
18	(2*E*,4*E*)-Hepta-2,4-dienal	1015	−	0.37 ± 0.03	1.02 ± 0.42	0.80 ± 0.10
19	2-Ethylhexan-1-ol	1033	−	−	−	0.89 ± 0.24
20	*p*-Cymene	1034	−	0.35 ± 0.17	−	−
21	Limonene	1037	0.60 ± 0.02	0.75 ± 0.25	−	0.68 ± 0.26
22	Benzyl alcohol	1042	2.22 ± 0.09	6.71 ± 0.12	1.60 ± 0.44	12.35 ± 0.88
23	(2*E*)-Oct-2-enal	1063	−	0.77 ± 0.01	1.29 ± 0.66	1.25 ± 0.17
24	(3*E*,5*E*)-Octa-3,5-dien-2-one	1097	−	1.20 ± 0.04		0.93 ± 0.03
25	Linalool	1102	−	0.22 ± 0.02	0.41 ± 0.15	0.53 ± 0.21
26	Nonanal	1107	−	0.89 ± 0.10	1.41 ± 0.55	2.20 ± 0.28
27	(2*E*,4*E*)-Octa-2,4-dienal	1114	−		1.36 ± 0.19	
28	2,6-Dimethylcyclohexanol	1115	−	0.61 ± 0.02		0.61 ± 0.14
29	6-[(*Z*)-1-Butenyl]-1,4-cycloheptadiene (Dictyopterene D)	1159	−	−	0.72 ± 0.28	−
30	(2*Z*)-Non-2-enal	1165	−	0.61 ± 0.11	−	2.47 ± 0.44
31	Indole	1297	−	0.27 ± 0.05	0.86 ± 0.34	0.37 ± 0.15
32	Tridecane	1300	0.93 ± 0.04	2.96 ± 0.39	0.71 ± 0.13	1.81 ± 0.06
33	α-Ionone	1433	2.63 ± 0.10	1.18 ± 0.01	2.52 ± 0.78	1.19 ± 0.29
34	Dimethyl phthalate	1461	−	0.44 ± 0.01	−	0.59 ± 0.04
35	(*E*)-β-Farnesene	1463	−	−	0.89 ± 0.19	0.31 ± 0.03
36	β-Ionone	1487	1.21 ± 0.05	0.61 ± 0.04	−	−
37	α-Curcumene	1488	−	−	0.58 ± 0.22	0.29 ± 0.03
38	Pentadec-1-ene	1495	−	0.51 ± 0.04	−	0.42 ± 0.08
39	Pentadecane	1500	0.48 ± 0.02	4.36 ± 0.01	0.47 ± 0.18	2.30 ± 0.48
40	5,6,7,7a-Tetrahydro-4,4,7a-trimethyl-2(4H)-benzofuranone	1534	0.39 ± 0.02	0.40 ± 0.07	−	−
41	Dihydroactinolide	1534	−	−	−	0.33 ± 0.13
42	Heptadecane	1700	6.65 ± 0.26	26.61 ± 0.90	11.22 ± 3.01	23.12 ± 1.86

SD—standard deviation.

**Table 2:** The volatile compounds from *C. adhaerens* isolated by hydrodistillation (HD) and analysed by gas chromatography–mass spectrometry (GC-MS): (VI—hydrodistillate of fresh *C. adhaerens*, VII—hydrodistillate of air-dried *C. adhaerens*).

No.	Compound	RI	Area (%) ± SD	
			VI	VII
1	Furan-2-carbaldehyde	<900	0.06 ± 0.01	0.11 ± 0.04
2	(2*E*)-Hex-2-enal	<900	0.36 ± 0.11	0.13 ± 0.05
3	4-Methyloctane	<900	0.15 ± 0.04	0.36 ± 0.19
4	Hexan-1-ol	<900	0.21 ± 0.06	−
5	Heptan-3-one	<900	0.07 ± 0.02	0.08 ± 0.00
6	5-Methylhexan-2-one	<900	−	0.31 ± 0.08
7	Non-1-ene	<900	0.05 ± 0.01	−
8	(2*E*,4*E*)-Hexa-2,4-diene	<900	−	0.04 ± 0.01
9	Heptanal	902	0.18 ± 0.04	0.51 ± 0.11
10	Hepta-2,4-dien-1-al *	907	−	0.07 ± 0.02
11	Diodomethane	923	−	0.16 ± 0.00
12	1-Iodopentane	927	−	0.08 ± 0.03
13	α-Pinene	941	−	0.03 ± 0.01
14	Benzaldehyde	966	0.40 ± 0.10	0.77 ± 0.14
15	Sabinene	979	−	0.03 ± 0.01
16	Oct-1-en-3-ol	985	0.10 ± 0.02	0.24 ± 0.02
17	Octan-2,3-dione	986	−	0.44 ± 0.16
18	Phenol	987	0.13 ± 0.04	0.08 ± 0.03
19	6-Methylhept-5-en-2-one	989	−	0.13 ± 0.04
20	2-Pentylfuran	996	0.21 ± 0.06	0.90 ± 0.04
21	2,4,6-Trimethylpyridine (α-Collidine)	997	0.09 ± 0.03	0.19 ± 0.09
22	Octanal	1004	0.10 ± 0.05	0.28 ± 0.06
23	(2*E*,4*E*)-Hepta-2,4-dienal	1015	0.08 ± 0.00	0.13 ± 0.05
24	2-Ethylhexan-1-ol	1033	0.11 ± 0.03	0.25 ± 0.09
25	Benzyl alcohol	1042	0.65 ± 0.14	0.67 ± 0.04
26	2,4,4-Trimethylcyclohex-2-en-1-ol	1059	0.15 ± 0.03	0.35 ± 0.01
27	(2*E*)-Oct-2-enal	1063	0.27 ± 0.06	0.33 ± 0.02
28	Acetophenone	1072	0.11 ± 0.03	0.17 ± 0.01
29	Octylcyclopropane	1074	0.19 ± 0.08	0.17 ± 0.06
30	Nonan-2-one	1095	−	0.07 ± 0.03
31	(3*E*,5*E*)-Octa-3,5-dien-2-one	1097	−	0.26 ± 0.01
32	Linalool	1102	0.13 ± 0.03	0.07 ± 0.02
33	Nonanal	1107	0.17 ± 0.02	0.25 ± 0.02
34	2,6-Dimethylcyclohexanol	1115	0.13 ± 0.03	0.28 ± 0.05
35	α-Cyclocitral	1122	−	0.03 ± 0.01
36	2-Hydroxy-3,5,5-trimethylcyclohex-2-en-1-one (2-Hydroxyisophorone)	1126	−	0.03 ± 0.01
37	4-Ketoisophorone	1150	0.18 ± 0.03	0.13 ± 0.02
38	(2*Z*)-Non-2-enal	1165	0.16 ± 0.03	0.22 ± 0.04
39	Benzylmethylsulfide	1171	−	0.20 ± 0.01
40	2,4-Dimethylbenzaldehyde	1180	−	0.09 ± 0.03
41	Decan-2-one	1196	−	0.48 ± 0.11
42	Safranal	1204	−	0.36 ± 0.06
43	Decanal	1209	0.14 ± 0.02	0.08 ± 0.04
44	β-Cyclocitral	1226	−	0.15 ± 0.05
45	Benzothiazole	1229	−	0.07 ± 0.02
46	β-Cyclohomocitral	1263	0.11 ± 0.04	0.11 ± 0.00
47	δ-Octalactone	1264	0.18 ± 0.03	−
48	(3*Z*)-Tridec-3-ene	1295	0.12± 0.03	−
49	1H-Indole	1297	0.30 ± 0.07	1.37 ± 0.19
50	Tridecane	1300	2.38 ± 0.52	1.04 ± 0.21
51	Undecanal	1311	0.14 ± 0.05	−
52	(2*E*,4*E*)-Deca-2,4-dienal	1321	0.15 ± 0.00	0.12 ± 0.03
53	1,1,6-Trimethyl-2*H*-naphthalene (3,4-Dehydroionene)	1358	0.09 ± 0.02	0.05 ± 0.02
54	Hexahydropseudoionone	1409	0.16 ± 0.03	0.08 ± 0.03
55	α-Ionone	1433	0.61 ± 0.12	5.84 ± 0.72
56	2-Methoxynaphthalene	1453	0.09 ± 0.03	0.05 ± 0.02
57	(*Z*)-Geranylacetone	1459	−	0.48 ± 0.06
58	(5*E*)-Dodec-5-en-1-ol	1467	0.23 ± 0.04	−
59	β-Ionone	1487	−	1.22 ± 0.45
60	β-Ionene	1490	0.85 ± 0.14	1.81 ± 0.33
61	Pentadec-1-ene	1495	2.12 ± 0.36	0.67 ± 0.12
62	Pentadecane	1500	3.25 ± 0.67	1.84 ± 0.27
63	(7*E*)-Pentadec-7-ene	1509	0.26 ± 0.04	−
64	Tridecanal	1515	0.21 ± 0.01	0.09 ± 0.03
65	Dihydroactinolide	1534	−	0.45 ± 0.08
66	Tetradecan-2-one	1566	0.21 ± 0.06	0.16 ± 0.01
67	Dodecanoic acid	1573	0.52 ± 0.06	0.19 ± 0.07
68	Tridecan-1-ol	1580	0.52 ± 0.02	0.37 ± 0.06
69	Diethyl phthalate	1599	−	0.32 ± 0.12
70	Hexadecane	1600	0.40 ± 0.15	0.14 ± 0.05
71	Tetradecanal	1617	0.54 ± 0.00	0.14 ± 0.03
72	Benzophenone	1630	−	0.23 ± 0.08
73	(6*Z*)-Dodec-6-en-4-olide ((Z)-6-γ-Dodecenolactone)	1661	2.46 ± 0.17	0.85 ± 0.09
74	Tetradecan-1-ol	1683	3.55 ± 0.38	2.16 ± 0.26
75	(8*E*)-Heptadec-8-ene	1690	0.44 ± 0.06	0.30 ± 0.11
76	Heptadec-1-ene	1697	2.80 ± 0.42	1.61 ± 0.13
77	Heptadecane	1700	29.32 ± 6.86	12.29 ± 0.25
78	(3*Z*)-Heptadec-3-ene	1709	0.32 ± 0.05	0.11 ± 0.04
79	Pentadecanal	1720	0.56 ± 0.03	0.17 ± 0.06
80	7-Methylheptadecane	1750	0.31 ± 0.00	0.70 ± 0.02
81	Tetradecanoic acid	1772	2.43 ± 0.39	0.24 ± 0.04
82	Octadec-1-ene	1790	0.19 ± 0.07	−
83	Octadecane	1800	0.29 ± 0.03	0.09 ± 0.03
84	Hexadecanal	1822	1.32 ± 0.08	0.19 ± 0.02
85	Neophytadiene	1845	0.16 ± 0.06	0.10 ± 0.04
86	Hexahydrofarnesyl acetone (Phytone)	1852	2.03 ± 0.10	5.81 ± 0.48
87	*p*-Cumylphenol	1857	0.27 ± 0.04	−
88	(9*Z*)-Hexadeca-1,9-diene	1866	0.49 ± 0.13	6.53 ± 0.48
89	(11*Z*)-Hexadec-11-enal	1870	0.21 ± 0.03	0.25 ± 0.04
90	Diisobutyl phthalate	1874	0.42 ± 0.02	1.10 ± 0.05
91	(9*E*)-Nonadec-9-ene	1879	0.49 ± 0.01	0.22 ± 0.03
92	Hexadecan-1-ol	1886	1.63 ± 0.25	1.57 ± 0.50
93	Nonadec-1-ene	1898	0.25 ± 0.04	0.10 ± 0.04
94	Nonadecane	1900	0.70 ± 0.08	0.33 ± 0.00
95	Hexadecanoic acid	1981	8.62 ± 1.61	10.55 ± 1.40
96	Cyclooctasulfur	2018	0.57 ± 0.05	0.42 ± 0.16
97	Octadecanal	2025	0.71 ± 0.08	0.22 ± 0.03
98	Methyl octadecyl ether	2034	2.06 ± 0.24	1.22 ± 0.08
99	Octadecan-1-ol	2086	0.51 ± 0.13	1.75 ± 0.90
100	*(E)*-Phytol	2119	5.00 ± 1.14	16.99 ± 2.69
101	(9Z)-octadec-9-enoic acid (Oleic acid)	2146	0.49 ± 0.27	2.53 ± 0.37
102	(5*E*)-Icos-5-ene	2293	0.65 ± 0.17	2.68 ± 1.32
103	4,8,12,16-Tetramethylheptadecan-4-olide	2383	−	0.33 ± 0.11

SD—standard deviation; *—correct isomer is not identified.

**Table 3:** Fatty acid composition of *C. adhaerens* determined by GC-FID.

No.	Fatty Acid	Av ± SD (%)
1	Dodecanoic acid (Lauric acid) (C12:0)	4.02 ± 1.16
2	Tetradecanoic acid (Myristic acid) (C14:0)	4.10 ± 0.28
3	Hexadecanoic acid (Palmitic acid) (C16:0)	25.50 ± 0.13
4	Octadecanoic acid (Stearic acid) (C18:0)	7.12 ± 0.26
5	Eicosanoic acid (Arachidic acid) (C20:0)	22.48 ± 0.49
6	Docosanoic acid (Behenic acid) (C22:0)	1.72 ± 0.19
	**Total saturated fatty acids (SFA)**	**64.94**
7	(9*Z*)-Hexadec-9-enoic acid (Palmitoleic acid) (C16:1)	5.79 ± 0.65
8	(10*Z*)-Heptadec-10-enoic acid (*cis*-Heptadecenoic acid) (C17:1)	1.96 ± 0.24
9	(9*Z*)-Octadec-9-enoic acid+(9*E*)-Octadec-9-enoic acid (*cis*-Oleic acid+*trans*-Oleic acid) (C18:1n9*c*+*t*)	16.91 ± 1.46
	**Total monounsaturated fatty acids (MUFA)**	**24.66**
10	(9*Z*,12*Z*)-Octadeca-9,12-dienoic acid (*cis*-Linoleic acid) (C18:2n6*c*)	4.67 ± 0.33
11	(9*Z*,12*Z*,15*Z*)-Octadeca-9,12,15-trienoic acid (α-Linolenic acid) (C18:3n3)	2.77 ± 0.41
12	(5*Z*,8*Z*,11*Z*,14*Z*)-Icosa-5,8,11,14-tetraenoic acid (Arachidonic acid) (C20:4n6)	1.78 ± 0.32
13	(13*Z*,16*Z*)-Docosa-13,16-dienoic acid (Docosadienoic acid) (C22:2n6)	1.63 ± 0.49
	**Total polyunsaturated fatty acids (PUFA)**	**10.85**
	**Total ω3 fatty acids**	**2.77**
	**Total ω6 fatty acids**	**8.08**

**Table 4:** Major non-volatile compounds in F3 and F4 fractions and their tentative identification by UHPLC-ESI(+)-HRMS.

No.	Compound	Rt (min)	Elemental Composition	*m*/*z* (Error, ppm)	Peak Area (Arbitrary Units)	
					F3	F4
1	Gingerglycolipid A	11.194	C_33_H_56_O_14_	677.37267 (2.379)	276,841.59	1288.25
2	2-Hydroxy-3-(β-L-talopyranosyloxy)propyl (9*Z*,12*Z*,15*Z*)-octadeca-9,12,15-trienoate	11.976	C_27_H_46_O_9_	515.32020 (2.445)	469,972.09	3546.75
3	1,3-Dihydroxy-2-propanyl icosa-5,8,11,14-tetraenoate	13.971	C_23_H_38_O_4_	379.28354 (1.972)	54,160.86	28,397.43
4	2,3-Dihydroxypropyl palmitate	14.222	C_19_H_38_O_4_	331.28296 (4.030)	377,335.75	495,766.09
5	2,3-Dihydroxypropyl octadec-9-enoate	14.561	C_21_H_40_O_4_	357.30074 (−2.247)	40,879.4	-
6	Isoamijiol oxidation product *	14.939	C_20_H_30_O_2_	303.23271 (−2.803)	994,098.63	18,848.2
7	2,3-Dihydroxypropyl stearate	15.254	C_21_H_42_O_4_	359.31454 (2.932)	505,462.75	659,353.94
8	2-Hydroxypropyl palmitate	15.379	C_19_H_38_O_3_	315.29087 (−4.761)	52,074.77	141,670.42
9	Pheophorbide *a*	15.537	C_35_H_36_N_4_O_5_	593.27486 (1.663)	1,478,183.5	175,460.75
10	Isoamijiol	15.569	C_20_H_32_O_2_	305.24830 (−2.598)	552,684.31	66,746.24
11	Fucoxanthin	15.6	C_42_H_58_O_6_	659.42919 (2.169)	23,608.59	20,924.63
12	(2*E*)-3-[21-(Methoxycarbonyl)-4,8,13,18-tetramethyl-20-oxo-9,14-divinyl-3,4-didehydro-3--24,25-dihydrophorbinyl]acrylic acid	15.632	C_35_H_30_N_4_O_5_	587.22619 (4.618)	16,648.76	94,242.22
13	3-[21-(Methoxycarbonyl)-4,8,13,18-tetramethyl-20-oxo-9,14-divinyl-3,4-didehydro-3--24,25-dihydrophorbinyl]propanoic acid	15.632	C_35_H_32_N_4_O_5_	589.24226 (3.887)	18,364.77	62,398.75
14	4-{[6-{[5-({[3-Carboxy-3-(dodecylamino)propanoyl]oxy}methyl)-3,4-dihydroxy-2-(hydroxymethyl)tetrahydro-2-furanyl]oxy}-3,4,5-trihydroxytetrahydro-2H-pyran-2-yl]methoxy}-2-(dodecylamino)-4-oxobutanoic acid	15.632	C_44_H_80_N_2_O_17_	909.55133 (1.815)	387,109.44	3907.02
15	13-Docosenamide	16.103	C_22_H_43_NO	338.34153 (0.637)	3958,621	859,522.19
16	2-Hydroxypropyl stearate	16.29	C_21_H_42_O_3_	343.32107 (−1.159)	114,486.16	343,931.75
17	3-(β-D-Galactopyranosyloxy)-2-[(7*Z*,10*Z*,13*Z*)-7,10,13-hexadecatrienoyloxy]propyl (9*Z*,12*Z*,15*Z*)-octadeca-9,12,15-trienoate	16.605	C_43_H_70_O_10_	747.50298 (1.590)	755,057.44	17,590.1
18	(3β,6α)-14-Methylergosta-8,24(28)-diene-3,6-diol	17.512	C_29_H_48_O_2_	429.37200 (1.646)	1,082,618.75	58,710.11
19	β-Stigmasterol	17.701	C_29_H_46_	395.36791 ** (−1.717)	58,667.22	514,958.41
20	1-Hydroxy-3-(tetradecanoyloxy)-2-propanyl (9*Z*)-9-octadecenoate	17.985	C_35_H_66_O_5_	567.49797 (0.580)	28,771.63	419,526.25
21	(3β)-3-Hydroxystigmast-5-en-7-one	18.364	C_29_H_48_O_2_	429.37170 (2.352)	440,882	287,291.56
22	3-Hydroxy-1,2-propanediyl bis(9-octadecenoate)	19.631	C_39_H_72_O_5_	621.54313 (3.412)	76,716.06	96,810.66
23	3-Hydroxy-2-(palmitoyloxy)propyl stearate	19.663	C_37_H_72_O_5_	597.54727 (−3.374)	134,634.83	139,876.53
24	Methyl 14-ethyl-4,8,13,18-tetramethyl-20-oxo-3-(3-oxo-3-{[(2*E*)-3,7,11,15-tetramethyl-2-hexadecen-1-yl]oxy}propyl)-9-vinyl-3,4-didehydro-24,25-dihydrophorbine-21-carboxylate	19.982	C_55_H_72_N_4_O_5_	869.55660 (1.098)	-	281,383.5
25	Methyl 9-acetyl-14-ethylidene-4,8,13,18-tetramethyl-20-oxo-3-{3-oxo-3-[(3,7,11,15-tetramethyl-2-hexadecen-1-yl)oxy]propyl}-13,14-dihydro-21-phorbinecarboxylate	20.011	C_55_H_74_N_4_O_6_	887.57206 (−4.454)	33,461.45	18,710,810
26	Methyl (10*Z*,14*Z*,20*Z*)-12-ethyl-3-hydroxy-13,18,22,27-tetramethyl-5-oxo-23-(3-oxo-3-{[(2*E*)-3,7,11,15-tetramethyl-2-hexadecen-1-yl]oxy}propyl)-17-vinyl-4-oxa-8,24,25,26-tetraazahexacyclo[19.2.1.1^6,9^.1^11,14^.1^16,19^.0^2,7^]hep-tacosa-1(24),2(7),6(27),8,10,12,14,16,18,20-decaene-3-carboxylate	20.013	C_55_H_74_N_4_O_7_	903.56436 (−1.473)	-	4582,960
27	Pheophytin *a*	20.168	C_55_H_74_N_4_O_5_	871.57287 (0.375)	-	45,562,208

*—exact compound not determined; **—dehydrated molecule [M-H_2_O+H]^+^.

**Table 5:** Antioxidant activity of *C. adhaerens* fractions (F3 and F4) obtained using ABTS assay with corresponding IC_50_ values and the significance parameters (confidence interval, slope and coefficient of determination, R^2^).

Sample	IC_50_ Value, mg/mL	Confidence Interval	Slope	R^2^
F3	2.44	1.94–3.64	1.36	0.997
F4	2.49	1.92–3.86	1.26	0.997

## Data Availability

Data is contained within the article.
